# Egypt: Its Artists, Intellectuals, and Neglected Tropical Diseases

**DOI:** 10.1371/journal.pntd.0005072

**Published:** 2016-12-01

**Authors:** Peter J. Hotez, Moustapha Kassem

**Affiliations:** 1 Sabin Vaccine Institute and Texas Children’s Hospital Center for Vaccine Development, National School of Tropical Medicine, Baylor College of Medicine, Houston, Texas, United States of America; 2 James A. Baker III Institute for Public Policy, Rice University, Houston, Texas, United States of America; 3 Department of Biology, Baylor University, Waco, Texas, United States of America; 4 Department of Endocrinology, University Hospital of Odense, Odense, Denmark; 5 Stem Cell Unit, Department of Anatomy, King Saud University, Riyadh, Saudi Arabia

Neglected tropical diseases (NTDs), the most common afflictions of the world’s poor, have been shown to exert important influences that extend beyond health. NTDs stifle economic development through their debilitating effects. They also stir up social stigma and cause severe psychological effects, especially for girls and women. There is an intimate link between NTDs and war or post-conflict situations, and, increasingly, with the recent emergence of Ebola and Zika, NTDs are further recognized as relevant to the global security agenda. We are still in the early stages of understanding the relationship between NTDs and such social forces, but one relationship seldom explored is the potential associations between NTDs and the arts and humanities. Possible illustrations of such a connection may be found in 20th century Egypt through the remarkable lives of Abdel Halim Hafez and Taha Hussein.

**Abdel Halim Hafez (1929–1977)** is ranked among Egypt’s greatest singers and artists. He was renowned and revered throughout the Arab world for his live performances and love songs. He was considered the Arabic “King of Emotions and Feelings” along with many other sobriquets, while influencing generations of singers and song writers (Fig 1) [1,2]. Abdel Halim Hafez also gave prominently to charities, and he had a long-standing friendship with Egyptian President Gamal Abdel Nasser [2]. When Abdel Halim Hafez died in 1977 at age 48 from liver failure due to schistosomiasis caused by *Schistosoma mansoni*, it was said that girls and women committed suicide, including some who "jumped off balconies" at his funeral march [1,2].

**Fig 1:**
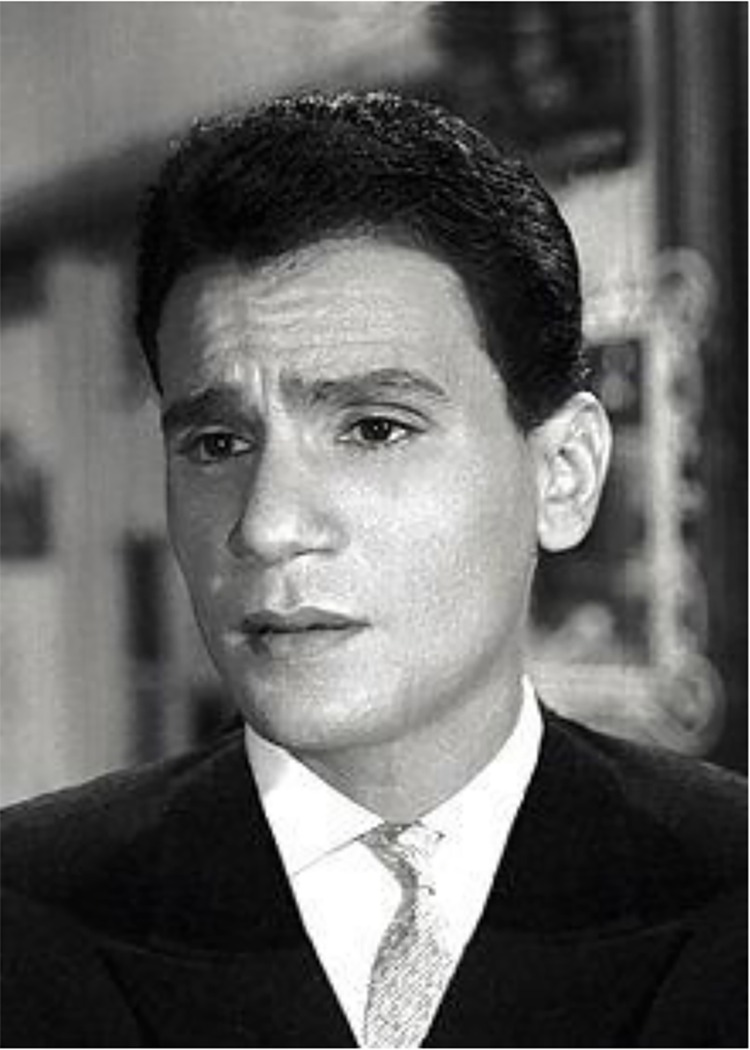
Abdel Halim Hafez. From https://en.wikipedia.org/wiki/Abdel_Halim_Hafez.

Abdel Halim Hafez grew up as an orphan, raised by an uncle and aunt who lived in extreme poverty [1,2]. For much of the 20th century, schistosomiasis was practically ubiquitous among the poor who lived along the Nile River and its tributaries [3]. This NTD was also difficult to treat before praziquantel became available. In its place, the injectable tartar emetic was used with horrific consequences due to the fact that hepatitis C virus regularly contaminated needles used to administer the drug [4]. As a result, hepatitis C and schistosomiasis coinfections became widespread and resulted in countless deaths from liver disease and failure [4].

Abdel Halim Hafez is believed to have contracted schistosomiasis as an adolescent [1,2], and he suffered from chronic liver disease due to the fibrosis that occurs from hepatic granulomas encasing schistosome eggs. He ultimately died from liver failure in a London hospital [2] where he was undergoing treatment that included portosytstemic shunt surgery in an attempt to reduce vascular pressures that resulted in esophageal varices. Attendance at his funeral may have reached millions, one of the largest ever in the Middle East and North Africa region [2].

**Taha Hussein (1889–1973)** was a contemporary of Abdel Halim Hafez, although he lived a much longer life, passing away in his 80s. He is generally considered Egypt’s greatest 20th century intellectual, spending much of his professional life at Cairo University, where he was considered the “Dean of Arabic Literature” (Fig 2) [5,6].

**Fig 2:**
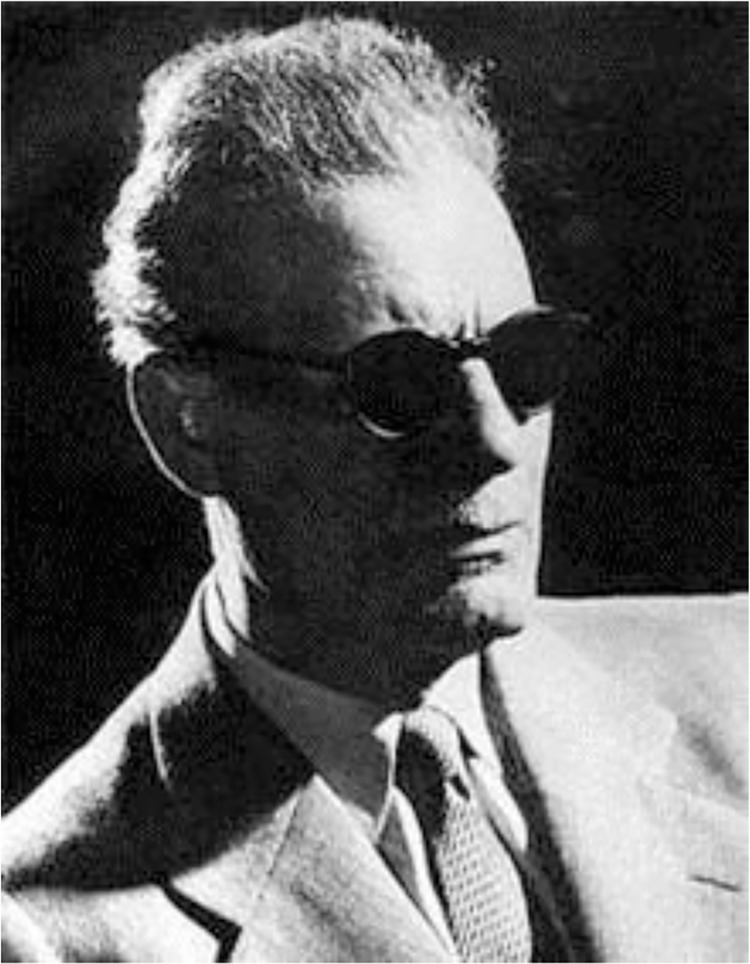
Taha Hussein. From https://en.wikipedia.org/wiki/Taha_Hussein.

Like Abdel Halim Hafez, Taha Hussein was born into extreme poverty and also contracted a debilitating NTD as a child. But, in Taha Hussein’s case, it was blinding trachoma acquired as a young child, possibly made worse by a surgical eye procedure performed by a village barber [5]. He recounts this experience in a renowned autobiography entitled *The Days*, published in 1929 and since republished in multiple languages [5,6].

Taha Hussein began his formal education in a religious school as an adolescent, but he was a prodigy, and his obvious brilliance allowed him to become one of the first matriculating students at the new Cairo University in 1908 [5]. He subsequently studied in Montpelier, where he met and married his lifelong companion, Suzanne Bresseau [5,6], and then went on to obtain another doctoral degree from the Sorbonne in Paris before finally coming back to Egypt for good to become a Cairo University professor.

Taha Hussein led an extraordinary intellectual life. Among his accomplishments, he founded two Egyptian universities, Alexandria and Ein Shams University, and edited numerous literary magazines [5]. His intellectual life was also turbulent, as he was removed on numerous occasions from leadership academic posts, only to be rehired [5]. He ultimately became Egypt’s Minister of Education in 1950, which earned him the designation of “Pasha” [5]. More importantly, he tireless devoted efforts to promote literacy across Egypt.

Whereas Abdel Halim Hafez was a close personal friend of Nasser (the president of Egypt between 1954–1970), such was not the case with Taha Hussein due to the latter’s liberal views [5]. Hussein wrote prolifically on almost all aspects of Egyptian and Arabic life and history, including Islamic history and commentary and the influence of Jewish scholars living in Arab lands [5]. Hussein was a strong proponent for modernization of the interpretation of the Islamic heritage and Islamic religious texts, as demonstrated in his famous book, *Pre-Islamic Poetry*, which was considered controversial and even banned for a time; he was even threatened with arrest [5]. He was also considered a great literary critic and theorist. Taha Hussein’s wife Suzanne was a lifelong friend and mother of their two children. Upon Taha Hussein’s death, she wrote a moving book about their literary marriage entitled *Avec Toi* (“With You”).

## Concluding Statement

It is difficult to say precisely how NTDs influenced the life and work of these two individuals. Possibly, in the case of Abdel Halim Hafez, his impoverished upbringing and experience with schistosomiasis helped to stimulate his charitable giving, including his establishment of a hospital [6]. In the case of Taha Hussein, the impact of his blindness from trachoma is detailed in *The Days*. Given that NTDs are practically ubiquitous among the poor and can produce horrific and debilitating effects, it leaves one to wonder how many artists and intellectuals through the ages were denied the chance to lead such productive and active lives.
